# Serum C1q/TNF-related protein-3 (CTRP3) levels are decreased in obesity and hypertension and are negatively correlated with parameters of insulin resistance

**DOI:** 10.1186/s13098-015-0029-0

**Published:** 2015-04-10

**Authors:** Wuquan Deng, Changyan Li, Yuping Zhang, Jie Zhao, Mengliu Yang, Mingyuan Tian, Ling Li, Yanling Zheng, Bing Chen, Gangyi Yang

**Affiliations:** Department of Endocrinology, the Second Affiliated Hospital, Chongqing Medical University, Chongqing, 400010 China; Department of Nephropathy, Chengdu Fifth Pepole’s Hospital, Sichuan Province, 611130 China; Department of Endocrinology, Southwest Hospital, Third Military Medical University, Chongqing, 400038 China

**Keywords:** C1q/TNF-related protein-3, Insulin resistance, Obesity, Essential hypertension

## Abstract

**Background:**

Insulin resistance (IR) correlates closely with cardiovascular disease. C1q/TNF-related protein-3 (CTRP3) is a novel adipokine that modulates insulin activity in various diseases. This study investigated the relationship between CTRP3 and IR as well as systemic inflammation in newly diagnosed obese and hypertensive patients (NCT02226471).

**Methods:**

Serum CTRP3 levels, anthropometric, inflammatory and metabolic parameters were measured in 180 obesity and essential hypertensive patients and in 66 normal weight, normotensive subjects.

**Results:**

The serum CTRP3 levels in the obesity group were lower than those in the NW group; these levels were also lower in hypertensive subjects than in normotensive subjects. After adjusting for gender, systolic blood pressure (SBP) and diastolic blood pressure (DBP), a modestly linear relationship was observed between CTRP3 and waist circumference (WC) (r = -0.168, p = 0.009), waist-to-hip ratio (WHR) (r = -0.183, p = 0.004), homeostasis model assessment of IR (HOMA-IR) (r = -0.264, p = 0.000), triglycerides (TG) (r = -0.136, p = 0.034), fasting blood glucose (FBG) (r = -0.155, p = 0.016), fasting insulin (FINS) (r = -0.248, p = 0.000) and homeostasis model assessment of β-cell insulin secretion (HOMA-β) (r = -0.128, p = 0.047). Multiple stepwise regression analysis revealed that gender, DBP and HOMA-IR were independently associated with serum CTRP3 levels.

**Conclusion:**

CTRP3 was an independent factor affecting blood pressure and IR, and may play an important role in the pathogenesis of obesity and hypertension.

## Introduction

Obesity has become a major global health problem, and the proportion of adults with overweight or obesity has increased substantially in the past thirty years [1]. Obesity is considered one of the most important risk factors of cardiovascular disease [2]. Previous research has indicated that more than two-thirds of diagnosed hypertension can be directly attributed to obesity [3]. Recent studies have demonstrated that adipokines play important roles in the pathogenesis of obesity and function as a link between obesity and obesity-related disorders [4]. Adipokines, such as adiponectin, resistin, retinol-binding protein 4 (RBP4), are secreted by adipose tissues, which may play a critical role in systemic inflammation and insulin resistance (IR) in obesity and hypertension. However, the precise roles of adipokines in regulation of IR require further investigation.

Recently, a new and highly conserved family of secreted proteins, C1q/tumour necrosis factor-related proteins (CTRPs), which includes fifteen family members, was shown to possess structural homologies to adiponectin. In addition, some CTRP members manifest the metabolic regulatory function of adiponectin. C1q/TNF-related protein-3 (CTRP3), a member of the CTRP superfamily, was identified as an anti-inflammatory adipokine that inhibits the inflammation induced by lipopolysaccharide, Toll-like receptor 4 and fatty acid [5] and induces adiponectin and resistin release in murine adipocytes [6]. Adiponectin is the most well-characterised member of the CTRP family, is tightly linked to IR and insulin sensitivity, and is an important biomarker and therapeutic target in obesity-associated metabolic diseases. To date, several CTRPs have been shown to induce insulin-sensitising effects similar to adiponectin [7]. Therefore, these proteins, such as CTRP3, might compensate for a deficiency of adiponectin, thereby providing an explanation for why adiponectin knockout mice display only mild metabolic disturbances, even when fed a high-fat diet [8].

To date, no data have been reported concerning the relationship between CTRP3 and drug-naive, newly diagnosed obesity and hypertension subjects. Therefore, we investigated the associations of CTRP3 and RBP4 with metabolic, inflammatory parameters and IR in present study.

## Subjects and methods

### Study population

We consecutively selected 826 Chinese subjects from the general population who had undergone medical check-ups at the Southwest Hospital Medical Center at the Third Military Medical University from March to November 2013. After excluding 580 of the 826 subjects, a total of 246 subjects were enrolled in our study. The first group enrolled 124 normal weight (NW) subjects. Their body weight status was confirmed by body mass index (BMI). The second group included 122 obesity subjects. All subjects were newly diagnosed and had not received any medical treatment. Obesity was defined according to the WHO Western Pacific Regional Office definition [9]. Based on blood pressure measurements, the subjects were divided into four subgroups: NW-normal blood pressure subgroup (NW-NBP), NW-hypertension subgroup (NW-HTN), obesity-normal blood pressure subgroup (OB-NBP), and obesity-hypertension subgroup (OB-HTN). The diagnosis of hypertension was based on the World Health Organization criteria [10].

The subjects exhibited stable body weight for at least 3 months prior to testing. The subjects enrolled in the analysis met the following inclusion criteria: (1) 20 years of age or older and younger than 65 years old; (2) essential hypertension; (3) obesity; and exclusion criteria: (1) fasting blood glucose (FBG) > 6.1 mmol/L and diabetes or thyroid disease; (2) taking antihypertensive, antidiabetic or lipid-lowering medications; (3) clinical symptoms or signs of infection; (4) liver, kidney, heart disorders or other critical diseases, such as fractures, osteoporosis or tumours; (5) target organ damage; (6) pregnant or lactating. The study was approved by the Human Research Ethics Committee at Southwest Hospital, and informed consent was obtained from all patients and controls.

### Anthropometric measurements and definitions

All participants were required to fast overnight (8-10 h) before the physical examination. Medical histories, anthropometric parameters were recorded followed by blood pressure measurements. Blood pressure was measured three times in 2 min intervals on the upper arm using a mercury sphygmomanometer (YuYue Medical Equipment & Supply CO., LTD., Jiangsu Province, China), with the subject in a seated, resting position after 10 minutes of rest; the mean of the three measurements was used for the statistical analysis. Anthropometric parameters, including weight, height and hip and waist circumference (WC), were measured using the International Collaborative Study on Hypertension in Blacks (ICSHIB) standardised protocol [11]. BMI was calculated as body weight in kilograms divided by the square of the height (m^2^). The waist-to-hip ratio (WHR) was calculated as the waist-to-hip circumference.

We defined current alcohol consumption as more than 1 drink of any type per month, and not currently drinking as less than 1 drink of any type per month [12]. Smokers were defined as having smoked more than 100 cigarettes in a lifetime and having smoked at least one cigarette daily for 6 months by the time of the interview [13]. Obesity was defined as a BMI ≥ 25 kg/m^2^, as suggested by the WHO Western Pacific Regional Office [9]. Hypertension was defined as a systolic blood pressure (SBP) ≥ 140 mmHg and/or a diastolic blood pressure (DBP) ≥ 90 mmHg [10].

### Experimental procedures and serum samples

Overnight fasting blood samples were collected in tubes containing liquid EDTA; the samples were centrifuged at -4°C and maintained at -80°C until assayed. A certified laboratory procedure was used to evaluate the metabolic parameters, including fasting blood glucose (FBG), blood uric acid (UA), total cholesterol (TC), triglycerides (TG), low-density lipoprotein C (LDL-C), high-density lipoprotein C (HDL-C) and fasting insulin (FINS). FBG was assayed using the glucose oxidase method. UA, TC, TG, LDL-C and HDL-C concentrations were determined enzymatically. The white blood cell (WBC) count was measured using the Serono-Baker System 9000 Hematology Analyzer (Serono-Baker Diagnostics, Allentown, Pennsylvania).

Serum CTRP3 levels were determined by enzyme-linked immunosorbent assay (human CTRP3 ELISA kit, Aviscera Bioscience, Inc., Santa Clara, CA, USA). The intra-assay coefficients of CTRP3 were 4-6%, and the inter-assay coefficients were 8-10%. The linear ranges of the assays were 15.6 to 1000 ng/ml for CTRP3. Serum RBP4 levels were evaluated using a commercially available ELISA kit (R&D Systems, Inc., Minneapolis, MN, USA). The intra-assay coefficients of RBP4 were less than 10%, and the interassay coefficients were less than 15%. The linear ranges of the assays were 1.0 to 300 μg/ml for RBP4.

The homeostasis model assessment of IR (HOMA-IR) as an indicator of IR and the homeostasis model assessment of β-cell insulin secretion (HOMA-β) were calculated from the FINS and FBG levels using the following equations: HOMA-IR = FINS (μU/ml) × FBG (mmol/l)/22.5; and HOMA-β = 20 × FINS (μU/ml) / FBG (mmol/l) - 3.5 [14].

### Statistical analysis

The experimental data were analysed using SPSS software (SPSS, Chicago, IL, USA), version 17.0. Continuous variables are presented as the means ± standard deviations, and categorical variables are presented as absolute and relative frequencies (%). Before the statistical analysis, the data were subjected to a normal distribution analysis using the Kolmogorov-Smirnov test. Non-normally distributed data (CTRP3, TG, FINS, HOMA-IR and HOMA-β) were converted by logarithmic transformation. Categorical variables were compared using the chi-squared test. The Student’s t-test and analysis of variance (ANOVA) were used to compare groups. In addition, a post-hoc least-significant difference (LSD) test was used for equal variances of assumed variables, and Dunnett's test was used for equal variances of variables that were not assumed. Interrelationships between variables were analysed with Pearson’s correlation analysis. Partial correlation analysis was performed to control for gender and blood preesure. Multiple regression analysis was performed using stepwise linear regression to correct the effects of the covariates and to test the independent factors, and p < 0.05 was considered to be statistically significant for all analyses.

## Results

### Clinical characteristics

The clinical characteristics of the study subjects are presented in Table 1. There were no significant differences among the four subgroups in terms of age, gender and LDL-C distribution. The levels of WHR, SBP, DBP, UA, TG, TC, WBC, HOMA-IR and HOMA-β were higher in the NW-HTN subgroup than in the NW-NBP subgroup, and the levels of WC, WHR, SBP, DBP, TG and HOMA-β were higher in the OB-HTN subgroup than in the OB-NBP subgroup. Compared with the NW-HTN subgroup, the levels of BMI, WC, WHR, UA, TG, FINS, HOMA-IR and HOMA-β were significantly increased in the OB-HTN subgroup. The levels of BMI, WC, WHR, SBP, UA, TG, HDL-C, FBG, FINS, WBC, HOMA-IR and HOMA-β in the OB-NBP subgroup and the levels of BMI, WC, WHR, SBP, DBP, UA, TG, HDL-C, FINS, WBC, HOMA-IR and HOMA-β in the OB-HTN subgroup were significantly increased compared with those in the NW-NBP subgroup.

**Table 1:** **Clinical characteristics of the study subjects**

**Factor**	**NW**		**OB**	
	**NW-NBP**	**NW-HTN**	**OB-NBP**	**OB-HTN**
Age (year)	49.21 ± 8.95	49.90 ± 8.63	49.28 ± 8.73	48.16 ± 7.87
Gender (M/F)^#^	66(43/23)	67(48/19)	58(35/23)	55(37/18)
BMI (kg/m^2^)	22.50 ± 1.45	22.10 ± 1.65	28.02 ± 1.65^ab^	28.52 ± 2.00^ab^
WC (cm)	79.88 ± 6.51	81.36 ± 7.02	91.80 ± 7.29^ab^	95.79 ± 5.76^abc^
WHR	0.87 ± 0.05	0.89 ± 0.05^a^	0.91 ± 0.06^a^	0.95 ± 0.05^abc^
SBP (mmHg)	119.26 ± 9.44	154.76 ± 11.39^a^	125.97 ± 10.09^ab^	159.89 ± 14.54^ac^
DBP (mmHg)	75.89 ± 6.61	100.07 ± 7.51^a^	77.97 ± 7.07^b^	101.65 ± 11.46^ac^
UA (μmol/l)	295.10 ± 72.89	332.22 ± 78.21^a^	346.61 ± 68.96^a^	387.85 ± 99.98^ab^
TG (mmol/l)	1.39 ± 0.60	2.05 ± 1.15^a^	2.05 ± 1.48^a^	2.41 ± 1.02^abc^
TC (mmol/l)	4.97 ± 0.92	5.38 ± 0.94a	5.03 ± 0.82b	5.25 ± 0.83
LDL-C (mmol/l)	2.70 ± 0.59	2.74 ± 0.58	2.80 ± 0.50	2.72 ± 0.49
HDL-C (mmol/l)	1.58 ± 0.40	1.41 ± 0.25	1.34 ± 0.28^a^	1.33 ± 0.34^a^
FBG (mmol/l)	5.38 ± 0.52	5.29 ± 0.54	5.60 ± 0.57^ab^	5.47 ± 0.47
FINS (pmol/l)	9.54 ± 3.23	10.93 ± 3.07	14.47 ± 6.13^ab^	16.13 ± 5.19^ab^
WBC (10^9^ cells/l)	5.25 ± 1.07	6.06 ± 1.17^a^	5.90 ± 1.50^a^	6.05 ± 1.50^a^
RBP4(μg /ml)	50.97 ± 15.19	65.09 ± 17.09^a^	49.76 ± 14.72^b^	64.08 ± 18.86^ac^
HOMA-IR	2.29 ± 0.85	2.60 ± 0.90^a^	3.64 ± 1.71^ab^	3.93 ± 1.34^ab^
HOMA-β	109.83 ± 51.11	130.62 ± 46.54^a^	145.83 ± 69.43^a^	175.19 ± 80.73^abc^
Smoking (%)^**#**^	32.8	37.0	33.6	33.6
Drinking (%)^**#**^	26.6	34.7	51.4^ab^	51.4^ab^

Data represent the means ± SD or frequency (percentage).

CTRP3, TG, FINS, HOMA-IR and HOMA-β were log-transformed prior to analysis.

^#^chi-squared test.

^a^p < 0.01 compared with NW-NBP.

^b^p < 0.01 compared with NW-HTN.

^c^p < 0.01 compared with OB-NBP.

NW, normal weight subject; OB, obese subject; NW-NBP, NW with normal blood pressure.

NW-HTN, NW with hypertension; OB-NBP, OB with normal blood pressure; OB-HTN, OB with hypertension.

BMI, body mass index; WC, waist circumference; WHR, waist-to-hip ratio.

SBP, systolic blood pressure; DBP, diastolic blood pressure.

UA, uric acid; TG, triglyceride; TC, total cholesterol.

LDL-C, low-density lipoprotein cholesterol; HDL-C, high-density lipoprotein cholesterol.

FBG, fasting blood glucose; FINS, fasting plasma insulin; WBC,white blood cell.

HOMA-IR, HOMA-IR index; HOMA-β, HOMA β cell function index.

### Serum CTRP3 and RBP4 levels

**Figure 1:**
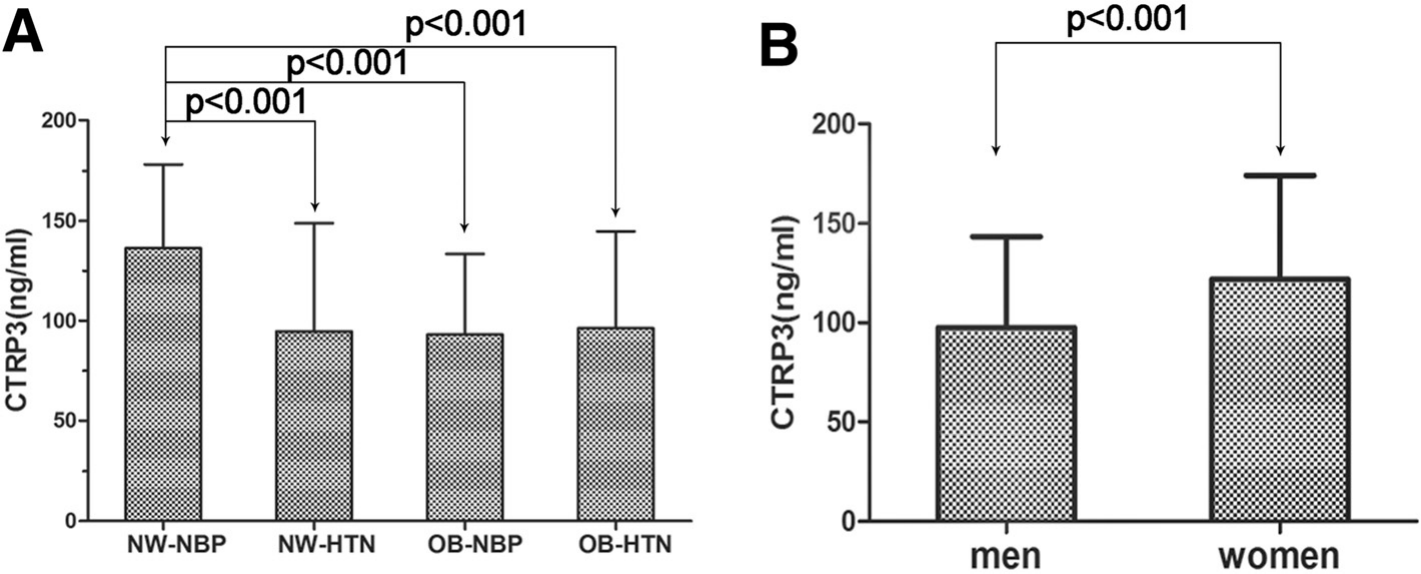
**The serum CTRP3 levels in different subgroups. A** - Comparison of the CTRP3 levels among the four subgroups. **B** - The serum CTRP3 levels in men and women.

The serum CTRP3 levels in the obesity group were lower than those in subjects with NW in both the NBP and HTN groups (94.53 ± 43.94 vs 116.84 ± 52.10 ng/ml, p < 0.001). Compared with the NW-NBP group, the serum CTRP3 levels were significantly decreased in the OB-NBP group (136.29 ± 41.86 vs 93.11 ± 40.26 ng/ml, p < 0.001). Compared with NW-NBP group, the serum CTRP3 levels were significantly decreased in the NW-HTN group (136.29 ± 41.86 vs 94.71 ± 54.05 ng/ml, p < 0.001) (Figure 1A). The CTRP3 concentration was significantly higher in women than in men (121.94 ± 52.23 vs 97.54 ± 45.92 ng/ml, p < 0.001) (Figure 1B).

There were no differences between the serum RBP4 levels in the normal weight and obesity subjects (57.58 ± 17.53 vs 56.22 ± 18.11 μg /ml, p = 0.550). Compared with the NW-NBP group, the RBP4 serum levels were significantly elevated in the NW-HTN group (50.97 ± 15.19 vs 65.09 ± 17.09 μg /ml, p < 0.001).

### Relationships between serum CTRP3 levels and metabolic, inflammatory as well as IR parameters

**Figure 2:**
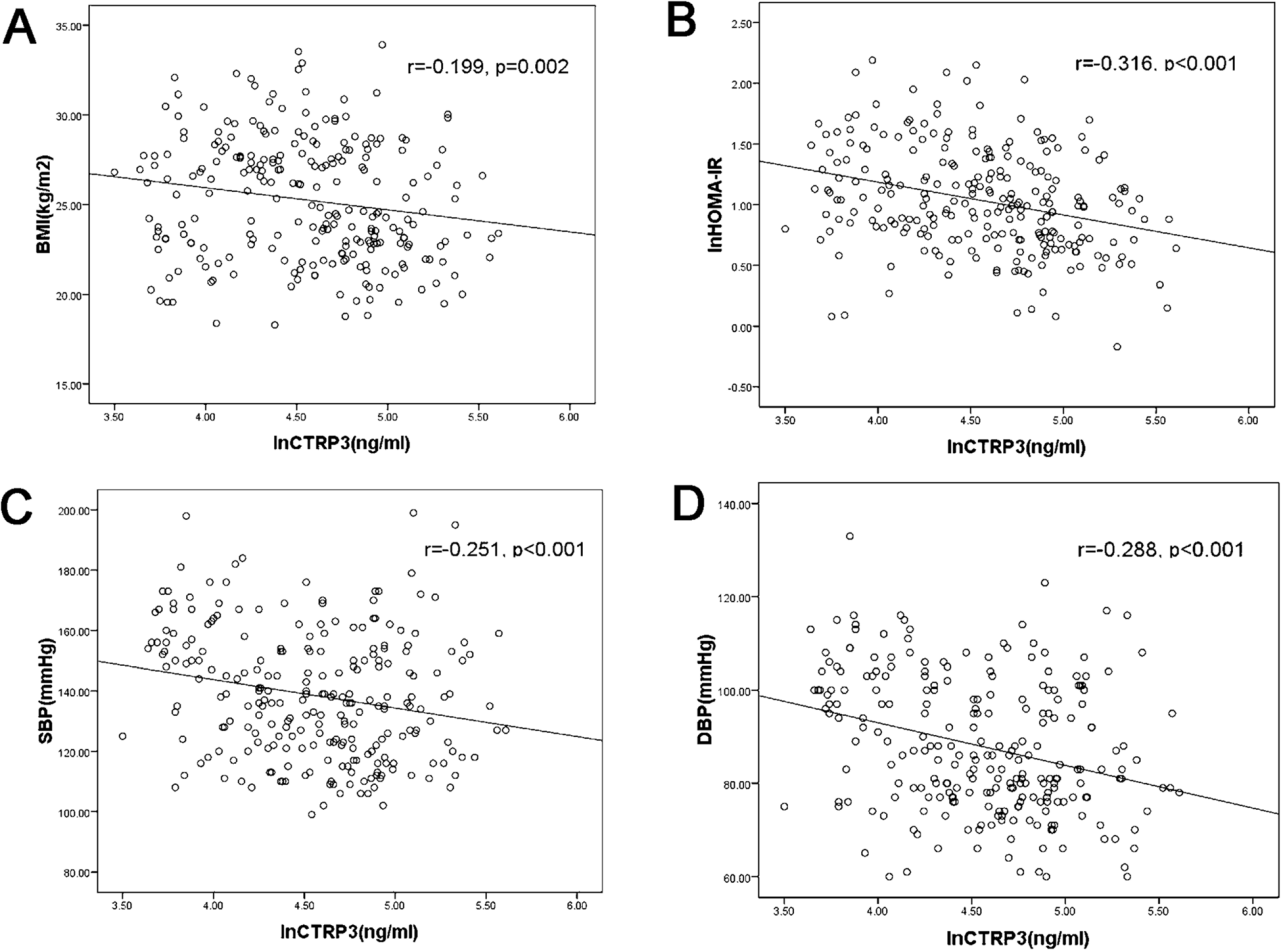
**Correlation between CTRP3 and BMI (A), HOMA-IR (B), SBP (C) or DBP (D).**

Bivariate correlation analysis indicated that serum CTRP3 concentrations were negatively correlated with body fat parameters, according to BMI (r = -0.199, p = 0.002) (Figure 2A), WC (r = -0.299, p = 0.000), WHR (r = -0.320, p = 0.000), HOMA-IR (r = -0.316, p = 0.000) (Figure 2B), as well as SBP (r = -0.251, p = 0.000) (Figure 2C), DBP (r = -0.288, p = 0.000) (Figure 2D), UA (r = -0.174, p = 0.006), TG (r = -0.241, p = 0.000), FBG (r = -0.137, p = 0.032), FINS (r = -0.308, p = 0.000), WBC (r = -0.170, p = 0.009), HOMA-β (r = -0.153, p = 0.003) and drinking (r = -0.150, p = 0.037). After adjusting for gender, SBP and DBP, the correlation remained between CTRP3 and WC (r = -0.168, p = 0.009), WHR (r = -0.183, p = 0.004), HOMA-IR (r = -0.264, p = 0.000), TG (r = -0.136, p = 0.034), FBG (r = -0.155, p = 0.016), FINS (r = -0.248, p = 0.000) and HOMA-β (r = -0.128, p = 0.047). After adjusting for gender, age and BMI, the correlation remained between CTRP3 and WC (r = -0.159, p = 0.013), WHR (r = -0.200, p = 0.002), HOMA-IR (r = -0.257, p = 0.000), TG (r = -0.168, p = 0.009), SBP (r = -0.296, p = 0.000), DBP (r = -0.288, p = 0.000), WBC (r = -0.152, p = 0.021), FINS (r = -0.250, p = 0.000) and HOMA-β (r = -0.140, p = 0.029). Multiple stepwise regression analysis revealed that gender, DBP and HOMA-IR were independently related to the CTRP3 levels (β = 0.159, -0.252, -0.240, all p < 0.05). The following multiple regression equation was used: Y _CTRP3_ = 5.425 - 0.009X_DBP_ - 0.303X _HOMA-IR_ + 0.164X_Gender_) (Table 2).

**Table 2:** **Linear and multiple regression analysis of the anthropometric and metabolic parameters associated with CTRP3 levels in the subjects**

	**Univariate correlations**		**Multivariate regression analysis**	
**Variable**	**r**	**p-value**	**β**	**p-value**
Gender	_	_	0.159	0.026
BMI (kg/m2)	-0.199	0.002	_	_
WC (cm)	-0.299	0.000	_	_
WHR	-0.320	0.000	_	_
SBP (mmHg)	-0.251	0.000	_	_
DBP (mmHg)	-0.288	0.000	-0.252	0.001
UA (μmol/L)	-0.174	0.006	_	_
TG (mmol/l)	-0.241	0.000	_	_
TC (mmol/l)	-0.111	0.081	_	_
LDL-C mmol/l)	-0.088	0.200	_	_
HDL-C (mmol/l)	0.125	0.068	_	_
FBG (mmol/l)	-0.137	0.032	_	_
FINS (pmol/l)	-0.308	0.000	_	_
WBC (10^9^ cells/l)	-0.170	0.009		
HOMA-IR	-0.316	0.000	-0.240	0.001
HOMA-β	-0.189	0.003	_	_
Smoking	-0.130	0.070	_	_
Drinking	-0.150	0.037	_	_
RBP4	0.065	0.313	_	_

CTRP3, TG, FINS, HOMA-IR and HOMA-β were log-transformed prior to analysis.

## Discussion

Adipose tissue secretes a variety of adipokines that play major roles in regulating glycolipid metabolism and systemic inflammation by directly or indirectly affecting insulin sensitivity and resistance [15]. CTRP3, a secreted protein, was cloned by Maeda et al. [16], first investigated in human plasma and reported by Wurm *et al*. [17]. Of the CTRPs studied thus far, CTRP3 is the closest functional homolog of adiponectin and the most promising candidate to play a role in inflammation and metabolism [7]. Compared with other adipokines, less information is known about CTRP3 in metabolic syndrome, systemic inflammation and IR, although the results of previous studies have been conflicting [18-21]. Therefore, it is worth exploring the clinical characteristics of CTRP3 in obesity and hypertension.

CTRP3 mRNA expression is strongly induced during adipocyte differentiation, and the knockdown of CTRP3 in preadipocytes leads to dedifferentiation into a more proinflammatory and immature phenotype [5]. Immunoblot analysis suggests that CTRP3 mRNA levels in fat tissue and protein levels in serum are similar in various mice models [22]. Circulating CTRP3 levels increase with fasting, are decreased in high-fat-diet-induced obese mice with high leptin levels, and are increased in leptin-deficient ob/ob mice [23]. CTRP3 may play an important role in progress of obesity through regulating energy metabolism, but mechanism should be further investigated. CTRP3 levels in metabolic syndrome and cardiovascular disease were puzzling in previous clinical studies. Recently, Tan et al. reported that serum and omental adipose tissue CTRP3 were significantly lower in women with polycistic ovary syndrome compared with control subjects, and a negative association was observed with BMI, WHR, glucose, insulin, and triglycerides, which is consistent with our results [20]. However, another research team reported that subjects with metabolic syndrome had significantly higher CTRP3 levels compared with subjects without metabolic syndrome; they also observed significant positive associations between plasma CTRP3 levels and cardiometabolic risk factors [18], inconsistent with Ban et al’s report recently [24]. A variety of reasons possibly were involved, such as ethnicity, medication and so on, in our study we excluded related confounding factors in order to reduce experiment divergence. Remarkably, the same researchers subsequently reported that CTRP3 levels were not significantly different in participants with and without obesity and that a 3-month combined exercise program significantly decreased CTRP3 levels in obese Korean women [19]. Interestingly, in another study, the same authors reported that serum CTRP3 was not significantly lower in subjects with metabolic syndrome compared with controls and that serum CTRP3 was significantly negatively associated with WC, DBP, serum glucose and triglycerides [21].

As we know, metabolic syndrome is composed of diverse components, including overweight or obesity, hyperglycemia, hypertension and dyslipidemia. We investigated the relationship between CTRP3 and individual component of metabolic syndrome, such as obesity and hypertension in order to understand more detailed each other after excluding other confounding factors. In the current study, we detected serum CTRP3 concentrations in humans and investigated the relationships between serum CTRP3 levels and relevant factors. The present data suggested that the CTRP3 concentrations were significantly decreased in patients with obesity or hypertension compared to control subjects. Moreover, a correlation analysis demonstrated that serum CTRP3 was significantly negatively associated with obese, inflammtionary and IR parameters as well as blood pressure levels. Multiple regression analysis revealed that only gender, DBP and HOMA-IR were predictive of serum CTRP3 levels. Serum RBP4 was considered as an inflammatory adipokine in early study, subsequent other research results were inconsistent, no significantly relationship was observed between RBP4 and CTRP3 in this study. Circulating RBP4 levels were not significantly different between obese patients and normal weight subjects but were significantly elevated in hypertensive patients compared with normotensive subjects, consistent with our previous study [25], which revealed that RBP4 possibly involved in pathogenesis of hypertension, not obesity.

Considering that adipokine expression may be affected by sex hormones, we compared serum CTRP3 concentrations in males and females, and the results showed that CTRP3 concentrations were significantly higher in women than in men. These sex-specific differences in CTRP3 levels were similar for adiponectin and other adipokines. The results of the current study are similar to a previous report [19]. Separately observating different genders, TG and HOMA-IR negatively influenced CTRP3 levels in both men and women subjectives, but the relation of FBG and FINS only was in women. Sex hormones may influence CTRP3 expression, it has been evidenced that androgen, oestrogen and adiponectin can regulate adiponectin receptors. CTRP3 was lower in sex hormones imbanlance women with polycystic ovary syndrome (PCOS) [20]. Animal experiments have also revealed that serum CTRP3 levels vary significantly according to the sex and genetic backgrounds of the mice [22].

Recent study indicates that CTRP3 is a potent anti-inflammatory adipokine that inhibits proinflammatory pathways. Ablation of CTRP3 by small interfering RNA increases the expression of proinflammatory adipokines including C-C motif chemokine ligand-2(CCL2) and decreases adiponectin expression in preadipocytes [5]. The WBC count is a widely available and broadly used marker of systemic inflammation. The present study revealed that CTRP3 levels were negative correlation with WBC in obese and hypertensive subjects. However, the relationship was vanished after adjusting for confounding fator of hypertension. The result indicated CTRP3 influences systemic inflammation possibly mainly in hypertensive, not obese subjects. The WBC count has been considered as a marker of cardiovascular disease risk measured through indicators of atherosclerosis and arterial stiffness in previous data [26].

CTRP3 mRNA was one of the most strongly up-regulated transcripts in rat carotid arteries after balloon injury [27]. In adult male mice, myocardial infarction significantly inhibits adipocyte CTRP3 expression and reduces plasma CTRP3 levels, and CTRP3 replenishment improves the survival rate, restores cardiac function, attenuates cardiomyocyte apoptosis, increases revascularisation, and dramatically reduces interstitial fibrosis, and the protective effect of adipocyte-conditioned medium against hypoxia-induced cardiomyocyte injury is significantly blunted when CTRP3 is knocked down [28]. A clinical study has suggested that coronary artery disease or stable angina pectoris patients have significantly lower circulating CTRP3 concentrations compared with control subjects, which suggests that CTRP3 may be useful in assessing the risk of coronary artery disease [29]. In the current study, serum CTRP3 was decreased and negatively correlated with blood pressure in hypertensive subjects, and one of independent predictor was DBP. Based on the above literature and results, CTRP3 as an anti-inflammatory cytokines has a cardioprotective effect through inhibiting production of pro-inflammatory cytokines, chemokines and so on. However, further mechanism is needed to clarify. In short, CTRP3 is a novel antiapoptotic, proangiogenic, and cardioprotective adipokine that may play an important role in directing vascular smooth muscle cell proliferation in the pathophysiology of neointimal hyperplasia and restenosis following angioplasty [30].

Adipokines can influence insulin sensitivity, and the regulation of adipokines may play a pivotal role in the aetiology of IR, which is associated with obesity and cardiovascular disease. Therefore, CTRP3 might also exert profound effects on local insulin sensitivity within adipose tissue. In this context, the present results the first time suggest that CTRP3 may indicate insulin sensitivity and plays an important and compensatory role in increasing insulin sensitivity and improving glycolipid metabolism. CTRP3 can sufficiently lower glucose levels and reduce glucose output in normal and insulin-resistant ob/ob mice by activating the Akt signalling pathway in the liver and suppressing the hepatic gluconeogenic gene and enzyme expression [23]. Similarly, Shike et al. described an association between fasting glucose levels and the centromeric region on chromosome 15 near D15Mit225, with a maximum score obtained in the region containing the CORS-26 gene [31].

## Conclusions

To the best of our knowledge, this study is the first to show that circulating CTRP3 levels are drastically decreased in naive, newly diagnosed obese and hypertensive patients. CTRP3, as an adipokine, was closely associated with IR, glycolipid metabolism and gender. Therefore, it may play a substantial pathophysiological role in the mechanism of obesity and hypertension. However, some study limitations must be considered. First, the study subjects consisted of a small number of men and women of Chinese ethnicity; hence, the generalisability of our study to other ethnicities is unknown. Second, this cross-sectional analysis restricts our ability to draw causal conclusions. Third, we did not perform oral glucose tolerance tests or glucose clamp studies to determine insulin resistance because of condition limitations. We observed close relationships between CTRP3 and IR, hypertension as well as sysmetic chronic inflammation, but the molecular mechanisms between these relationships require validation in future studies.
